# Concomitant Case of Anti-Glomerular Basement Membrane (GBM) Antibody Disease and Membranous Nephropathy

**DOI:** 10.7759/cureus.56672

**Published:** 2024-03-21

**Authors:** Chetan U Phadke, Shreeharsh S Godbole, Atul D Sajgure, Tushar A Dighe, Charan B Bale

**Affiliations:** 1 Nephrology, Dr. D. Y. Patil Medical College, Hospital & Research Centre, Dr. D. Y. Patil Vidyapeeth, Pune, IND

**Keywords:** nephrology disorders, plasmapheresis treatment, glomerulonephritis, membranous nephropathy, anti-gbm ab disease

## Abstract

Anti-glomerular basement membrane (GBM) disease is a form of rapidly progressive glomerulonephritis with acute deterioration of kidney function. Atypical forms of this disease have been described which do not show positive serology for the classical anti-GBM antibody (Ab) but their presence on kidney biopsies. Furthermore, concomitantly any other separate glomerular pathology along with anti-GBM disease has been only rarely seen. A 40-year-old male patient presented with complaints of lower limb swelling and hematuria. Initial blood investigations revealed nephrotic range proteinuria and hypoalbuminemia. The patient underwent a renal biopsy. Initial reports showed the presence of “linear” deposits for immunoglobulin G (IgG) Ab and crescent formation in the majority of glomeruli. Treatment with plasmapheresis was initiated for the same. Electron microscopy, which later revealed subepithelial deposits raised suspicion of concomitant membranous nephropathy (MN). This finding was confirmed with a staining biopsy block with an anti-PLA2R Ab stain. Treatment was initiated to treat both glomerular pathologies, which very rarely present together and do not have standard guidelines for treatment. The patient responded to treatment with a reduction in serum creatinine values and did not require maintenance hemodialysis. There have been only a handful of documented cases, only in the form of a few case series that have described the presence of both anti-GBM disease and MN in the same kidney biopsy.

## Introduction

Anti-glomerular basement membrane (GBM) disease is an autoimmune disease affecting the glomerulus and is known to develop in genetically susceptible individuals exposed to some environmental factors. It is characterized by linear deposition of immunoglobulin G (IgG) along the GBM. Anti-GBM disease is histologically associated with extensive crescent formation and clinically with rapidly progressive glomerulonephritis (RPGN) [1]. Sometimes concurrent occurrence of other diseases, such as anti-neutrophil cytoplasmic antibody (ANCA)-associated vasculitis, can lead to related glomerular injury. [2]. Membranous nephropathy (MN) is a form of kidney disease that is idiopathic in 70%-80% of cases. The remaining cases are associated with other conditions, ranging from infections, autoimmune diseases, neoplasms, and medications (secondary MN). It is the most common cause of nephrotic syndrome in the elderly, characterized by subepithelial immune complex deposition along the GBM [3]. There have been only a handful of reports, published as single case reports or short case series, where patients have presented with anti-GBM antibodies and MN. There is no defined clinical feature or standardized algorithm in therapy for such cases.

## Case presentation

A 40-year-old male patient presented at the nephrology OPD with complaints of swelling in both lower limbs for the past six months. The swelling had gradually increased over time and had now reached his knees from his ankles within the last seven days. The patient also reported a reddish tinge of urine 30 days before this presentation, and foamy urine for the past seven days. This had not happened previously over six months.

On further questioning, the patient gave a history of having had two to three episodes of blood-tinged sputum with a cough six months ago. The patient was extensively worked up (X-ray chest and cartridge-based DNA test of sputum) at that time for pulmonary tuberculosis but the tests had been negative for *Mycobacterium tuberculosis* infection. He also was diagnosed to have low hemoglobin during that evaluation and had received 2 pints of packed cell volume (PCV) transfusion. The anemia had not recurred and he did not receive any blood transfusions subsequently.

During his routine follow-up three months later he was found to be HBsAg positive. This was attributed to the blood transfusions the patient had received three months ago, as there was no history of any illicit intravenous drug abuse or any extramarital unprotected sexual activity. The patient had a known case of hypothyroidism and had been on thyroid hormone replacement for four years (current dose of L-thyroxine 25 micrograms/ day).

The patient had no history of hypertension, diabetes, ischemic heart disease, tuberculosis, bronchial asthma, trauma to his legs, or renal calculi. He had not undergone any minor or major surgical procedures in the past. The patient was physically examined. The findings are mentioned in Table 1.

**Table 1:** Vitals and examination findings at admission BP - blood pressure, SpO2 - oxygen saturation, CVS - cardiovascular system, RS - respiratory system, PA - posteroanterior, CNS - central nervous system

Parameter	Value
Pulse	100/min
BP	148/88 mmHg
SpO2	98% on room air
Pedal edema	Extending on both legs up to knees
CVS	S1,S2 heard normally, no murmurs
RS	Air entry equal on both sides
PA	Soft, non-tender, no obvious organomegaly
CNS	Conscious, alert, oriented, no obvious focal deficits

The patient was admitted to the nephrology ward for further examination and investigations. Investigations on admission are mentioned in Table 2.

**Table 2:** Laboratory investigations on admission HPF - high power field, UPCR - urine protein creatinine ratio, SGOT - serum glutamic-oxaloacetic transaminase, SGPT - serum glutamic pyruvic transaminase, ALP - alkaline phosphatase, C3 - complement 3, C4 - complement 4, c-ANCA - cytoplasmic antineutrophilic cytoplasmic antibody, p-ANCA - perinuclear antineutrophilic cytoplasmic antibody, T3 - triiodothyronine, T4 - thyroxine, TSH - thyroid stimulating hormone

Test	Result	Laboratory reference range
Hb	8.3 gm%	13.2-16.6 gm%
Total leucocyte count	10700/cumm	4000-10000/cumm
Platelet	2.75 lakh/cumm	1.5-4.1 lakh/cumm
Urea	55 mg/dL	17-49 mg/dL
Creatinine	5.51 mg/dL	0.6-1.35 mg/dL
Sodium	124 mmol/L	136-145 mmol/L
Potassium	3.7 mmol/L	3.5-5.1 mmol/L
Urine routine	Protein - 2+	Absent
	RBC - 12-15 per HPF	1-2 per HPF
	PC - 1-2 per HPF	1-2 per HPF
UPCR	17.43 gram/gram	<0.20 g/g
Total bilirubin	0.34 mg/dL	0.22-1.2 mg/dL
Direct bilirubin	0.11 mg/dL	<0.5 mg/dL
SGOT	14 U/L	8-48 U/L
SGPT	14 U/L	7-55 U/L
ALP	53 U/L	40-129 U/L
C3	85 mg/dL	90-120 mg/dL
C4	27 mg/dL	10-40 mg/dL
ANA Blot (17 antigens)	Negative	-
c-ANCA	Negative	-
p-ANCA	Negative	-
T3	0.96 ng/mL	0.64-1.52 ng/mL
T4	8.02 microgram/dL	4.87-11.72 microgram/dL
TSH	1.17 microIU/dL	0.35-4.94 microIU/dL

USG of the abdomen and pelvis showed no significant abnormalities. The right kidney measured 107x41 mm and the left kidney measured 121x60 mm with maintained cortico-medullary differentiation and raised echogenicity. There was no evidence of any obstruction or dilation of the pelvicalyceal system.

**Figure 1:**
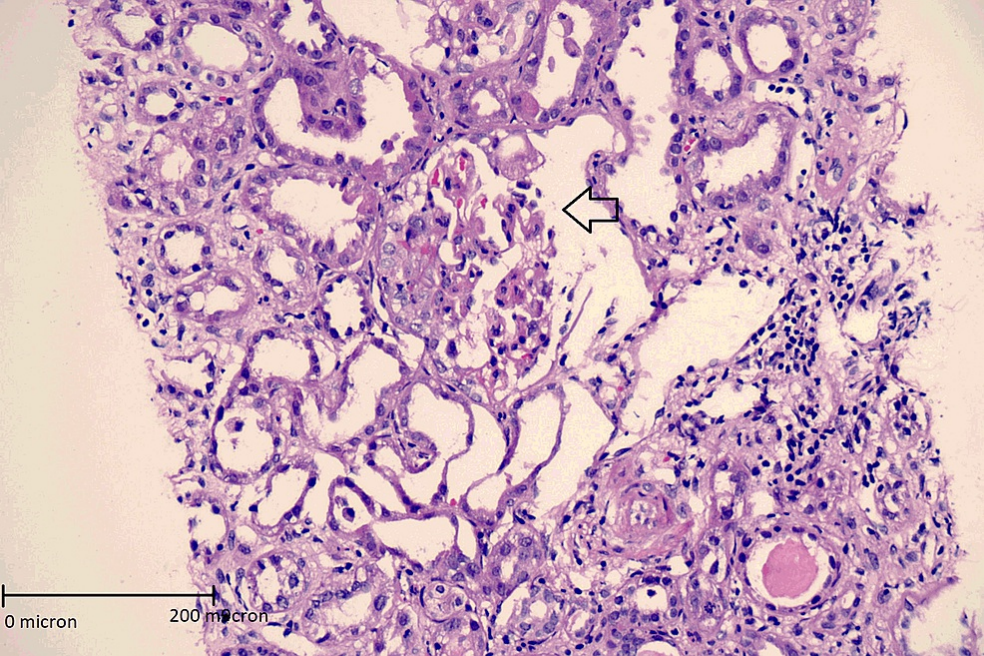
Hematoxylin and eosin stained image of kidney biopsy specimen showing crescent formation with rupture of Bowman’s capsule (marked with arrow) 400x magnification

With the above laboratory investigations and radiological investigations in mind, a renal biopsy was planned for the patient. The patient underwent a renal biopsy on light microscopy. Seventeen glomeruli were seen. Eight glomeruli showed partial small crescents (four cellular, one fibro-cellular, and three fibrous). Three glomeruli showed fibrinoid necrosis. Glomeruli also showed endothelial cell edema and prominent and activated visceral epithelial cells showed occasional mitotic figures. Tubular atrophy and interstitial fibrosis involved about 25-30% of the sampled cortex. Arteries showed subintimal edema and focal necrosis in the vessel wall, while arterioles showed subintimal edema and thickened walls. Figure 1 depicts a crescent and rupture of the glomerulus.

The immunofluorescence of the sample kidney was negative for IgA, IgM, C3, and C1q, while IgG was 3+ with linear deposition along capillary walls; of which IgG1 was 3+ linear staining along capillary walls and IgG2, IgG3, IgG4 were all negative.

**Figure 2:**
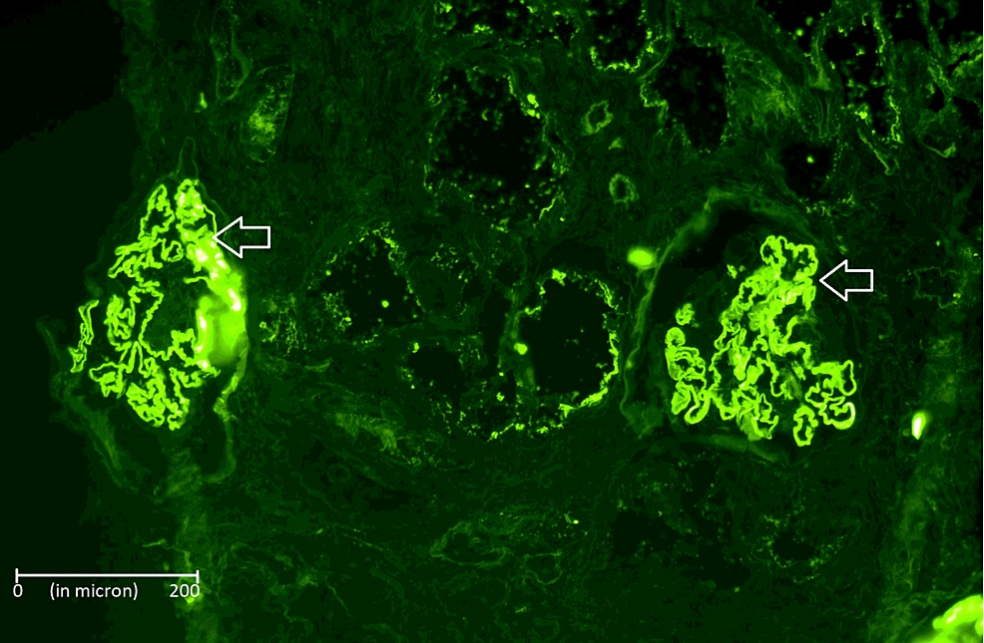
Immunofluorescence of kidney biopsy image demonstrating IgG deposits in linear deposits (shown with arrows) 400x magnification

Kappa light chains and Lambda light chains showed 3+ linear staining along capillary walls. Figure 2 shows the immunofluorescence image.

**Figure 3:**
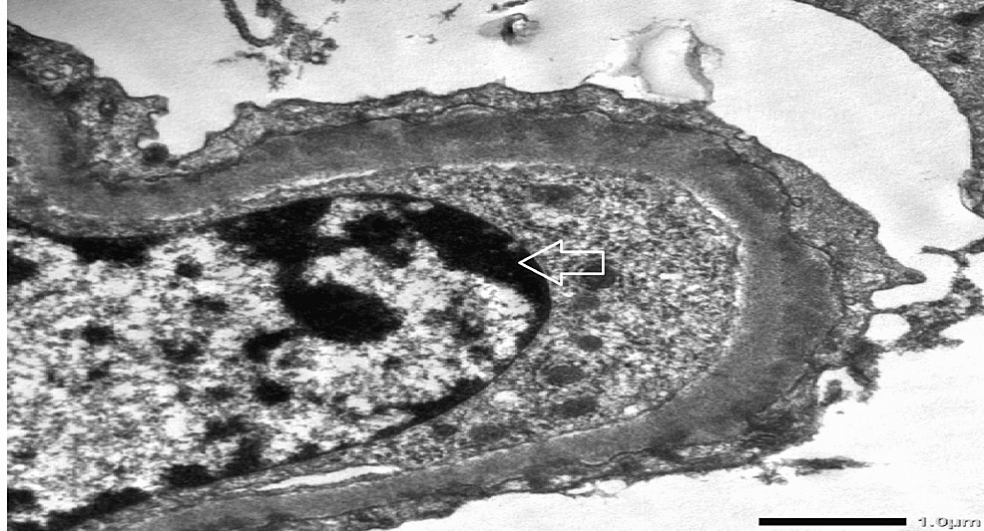
Electron microscopy demonstrating electron-dense deposits present in the subepithelial areas (shown with arrow)

The electron microscopy report was finalized and it showed two glomeruli. Both appeared normocellular and revealed foot process flattening of 95 percent of the loops. Both glomeruli revealed sub-epithelial electron-dense deposits. In certain regions, they impinged upon the lamina densa. A few loops revealed wrinkling. No crescents were seen in these glomeruli. No sclerosis or organized deposits were detected. Figure 3 shows the electron microscopy image of the glomerular basement membrane.

The kidney tissue was subjected to anti-PLA2R antibody and showed weak staining along glomerular capillary walls.

The anti-GBM Ab assay turned out to be negative, and consideration of a possibility of “atypical anti-GBM nephritis” was made. A final diagnosis of “MN with superimposed anti-GBM disease (supported by linear staining of IgG)” was made. 

The patient had initially presented with symptoms suggestive of a mixed nephrotic and nephritic picture. History of hemoptysis six months ago was not considered relevant to the current presentation, which was attributed to a possibility of *Mycobacterium tuberculosis* which is common in India (anti-tubercular treatment was not given as there was no evidence of the disease found).

The presence of positive serology for hepatitis B with nephrotic range proteinuria (>3g/ 24 hours) brought a differential of secondary membranous nephropathy disease into the picture. The possibility of focal segmental glomerulosclerosis (FSGS) was also considered in view of proteinuria, hematuria, and renal dysfunction. The renal biopsy on light microscopy was suggestive of anti-GBM disease and the serology for the same also was negative, and thus a diagnosis of atypical anti-GBM disease was made [4]. The hemoptysis could have been a symptom of the anti-GBM disease which was not considered at the time (chest X-ray was normal).

The patient was initiated on inj. cyclophosphamide pulse at 1.5 mg/kg and pulse inj. methylprednisone 500 mg IV for three days according to KDIGO (Kidney Disease: Improving Global Outcomes) guidelines. The patient also underwent three sessions of plasmapheresis. The Patient was continued on oral steroids at 1mg/ kg and oral cyclophosphamide. Subsequently, the patient at follow-up one month later had reduced proteinuria with urine protein creatinine ratio (UPCR) of 3.1 g/g and serum creatinine of 3.6 mg/dL. The patient’s pedal edema had subsided and there was no hematuria. The patient’s kidney function had recovered and did not require renal replacement therapy.

## Discussion

Since the first description of MN and anti-GBM disease in 1974 by Klassen et al. only a few case reports have been published [5]. Most patients were treated with corticosteroids, immunosuppressive drugs, and plasmapheresis. However, favorable outcomes were only achieved in three cases [5,6]. The rapid decline in renal function may occur in membranous nephropathy patients with additional superimposed factors such as severe hypertension, renal vein thrombosis, crescentic glomerulonephritis, and anti‐GBM mediated disease [7]. A phenomenon of anti-GBM Ab disease with negative serology has also been described in the literature [8]. Patients usually present with a distinct set of signs and symptoms. Fewer patients present with oliguria and anuria, crescent formation but significantly higher proteinuria, which is attributable to the MN [9]. Most reported cases of combined anti-GBM nephritis and MN were phospholipase A2 receptor-negative, as was the case in our patient [10]. Patients with MN with anti-GBM disease have distinct clinical outcomes compared to primary anti-GBM disease. In all reported cases of MN with anti-GBM disease, the prognosis has been poor despite immunosuppressive therapy, with progression to end-stage renal disease (ESRD) or death [11]. Savige et al. performed a study where they observed six patients who had both anti-GBM disease and membranous nephropathy [12]. These patients were treated with steroids, cyclophosphamide, and plasmapheresis. Out of the six patients, five were aged between 15-22 years and showed good recovery, while one patient who was 47 years old remained dependent on dialysis. Notably, younger patients exhibited hematuria and/or hemoptysis as the presenting symptoms, whereas middle-aged patients showed edema consistent with membranous glomerulonephritis, followed by anti-GBM disease. The proteinuria levels ranged from trace to 20.7 gm/24 hr, depending on the extent of renal damage [12]. 

Anti-GBM disease is a rare autoimmune disorder that can affect the kidneys. The pathogenesis of this disease involves immunologic mechanisms that can lead to the development of membranous-type deposits in the glomerular basement membrane. This can cause the release of normal or altered glomerular basement material, which can trigger the development of anti-GBM antibodies and crescentic glomerulonephritis. Studies on brown Norway rats have shown that exposure to mercuric chloride can induce autoimmune disease and cause the formation of autoantibodies directed against multiple basement membrane proteins and proteoglycan components. Understanding the immunologic mechanisms involved in anti-GBM disease is important for developing effective treatments for this rare condition [13]. Anti-GBM disease is a condition where antibodies are produced against a particular protein present in the kidneys called glomerular basement membrane (GBM). Two mechanisms can lead to the formation of these antibodies. Firstly, in situ immune complex formation can occur leading to membranous deposits, which can cause injury to podocytes and increase antigen synthesis. Secondly, anti-GBM antibodies can arise after damage to the glomerular basement membrane by vasculitis involving the glomerular capillaries. This damage can uncover hidden antigens, leading to the formation of antibodies against GBM. Anti-GBM disease can also occur after primary glomerulonephritis such as membranous nephropathy or IgA glomerulonephritis [14].

## Conclusions

To conclude, patients presenting with hemoptysis should be extensively evaluated for non-infective causes. The possibility of atypical anti-glomerular basement membrane (GBM) disease should still be considered in patients with negative serology of antibodies to GBM. Blood tested for serologies of infections transmissible by blood can still be positive for the infections. The patient may become positive later on, which is a known risk factor for the development of membranous nephropathy. Secondary causes of membranous nephropathy should be considered in patients with positive serologies of hepatitis B which are well documented in the literature.
